# Prophylactic Cranial Irradiation prior to HCT for Acute Lymphoblastic Leukemia: To Boost or Not To Boost

**DOI:** 10.46989/001c.124270

**Published:** 2024-10-07

**Authors:** Khalid Halahleh, Mohammad S. Makoseh, Ayat M. Taqash, Fawzi Abuhijla, Lubna S. Ghatasheh, Rozan B. Al Far, Lina M. Wahbeh, Isra F. Muradi, Abdelatif M. Almousa, Ramiz A. Abu-Hijlih, Hasan Hashem

**Affiliations:** 1 Internal Medicine, Bone Marrow Transplantation and Cellular Therapy Program King Hussein Cancer Center; 2 Internal Medicine-BMT Program and Cellular therapy King Hussein Cancer Center; 3 Research Office King Hussein Cancer Center; 4 Radiation Therapy King Hussein Cancer Center; 5 Department of Pediatrics King Hussein Cancer Center; 6 Internal Medicine, BMT and Cellular Therapy program King Hussein Cancer Center; 7 Internal Medicine University of Tripoli; 8 Radiation Therapy King Hussien Cancer Center; 9 Department of Pediatrics, Pediatric Bone Marrow Transplantation and Cellular Therapy Program King Hussein Cancer Center

**Keywords:** Acute lymphoblastic leukemia, allogeneic hematopoietic cell transplantation, conditioning, total body irradiation, cranial radiation boost, central nervous system relapse

## Abstract

**Background:**

Total body irradiation (TBI) with or without cranial radiation boost (CRB) is an integral component of conditioning prior to allogeneic hematopoietic cell transplantation (allo-HCT) in acute lymphoblastic leukemia (ALL). The benefit of CRB is not yet established.

**Methods:**

This is a retrospective single center cohort study. Between January of 2003 and April of 2019, electronic medical records of 166 patients with ALL were retrospectively reviewed. One hundred forty-three patients with ALL and no prior central nervous system (CNS) involvement were included. Patients were divided into two cohorts according to cranial radiation boost (cohort-1: CNS-/CRB+ (110/143, 77%) and cohort-2: CNS-/CRB- (n=33/143; 23%). No patients received post-transplant prophylactic intrathecal chemotherapy.

**Results:**

Following alloHCT, 15 patients (10.5%) experienced relapse; 11 relapses (10%) in cohort-1, and 4 (12%) in cohort-2. Four patients (26.6%) experienced systemic medullary relapse with initial central nervous system (CNS) involvement. One patient (6.6%) experienced isolated first central nervous system relapse after allotransplant with no difference between the two cohorts (6.6% vs 0; P-0.59). Age at transplant and phenotypic subtype were predictive of first central nervous system relapse after allotransplant with respective P-values of 0.001 and 0.015.

At a median follow-up of 30 months (range: 2.5-128 months), the estimated 3-year overall survival was 61% (95% CI: 53-69), relapse free survival was 60% (95% CI: 52-69) and 3-year central nervous system-relapse-free survival was 99% and 100% in in cohort-1 and cohort-2 respectively, when systemic relapses were censored. There was no statistical significant difference in either survival or relapse free survival between the two cohorts (P > 0.69).

**Conclusions:**

Our results suggest that augmenting total body irradiation with cranial radiation boost in patients with ALL with no prior CNS involvement did not improve relapse risk in central nervous system or survival outcomes.

## Introduction

Over the last decade, there has been tremendous progress in the treatment of acute lymphoblastic leukemia (ALL).^1^ Allogeneic hematopoietic cell transplantation (allo-HCT) is the current standard of care in the management of high-risk and relapsed ALL. Several randomized trials have demonstrated survival advantage of allo-HCT consolidation in high-risk adult and pediatric patients with ALL.^2,3^ Total body irradiation (TBI) has been widely used as an integral part of the conditioning regimen in ALL, because of eminent immunosuppressive and antileukemic effects, and the ability to treat sanctuary sites, such as the central nervous system (CNS).^4^

Synchronous medullary and CNS involvement is uncommon at diagnosis (5%); CNS is the most common extramedullary site of relapse.^5,6^ With modern ALL therapies, CNS relapse occurs in 4- 8% of patients treated with combination chemotherapy.^7,8^ In previously published reports, CNS involvement, either at diagnosis or at subsequent relapse, predicts a higher risk of post-transplant CNS relapse, while other reports found no significant correlation.^4–6,9–11^ Several strategies have been employed in the peri-transplant period to prevent CNS relapse, including the use of TBI-based conditioning, and post-transplant prophylactic intrathecal chemotherapy.^12–14^ Some institutions have integrated CRB into the TBI-based conditioning regimen in high-risk ALL patients, although the efficacy of this approach has yet to be established, and the data are scarce and conflicting.^9–15^ The aim of this study is to determine the effect of CRB prior to alloHCT on posttransplant first CNS relapse and survival outcomes in patients with ALL without CNS involvement at the time of diagnosis.

## Materials and Methods

### Patient’s characteristics

This is a retrospective single center cohort study of 166 consecutive patients with ALL, who received their first allo-HCT at the adult and pediatric bone marrow transplant programs, at King Hussein Cancer Center (KHCC) between January of 2003 and April of 2019. All patients with ALL, who received TBI-containing preparative regimen were identified from the Blood and Marrow Transplantation KHCC Database, which prospectively collects baseline patient’s characteristics and transplantation details, in addition to disease, treatment complications, and outcome data. All patients were in complete morphological remission (CR) at the time of transplantation. The study was approved by the KHCC institutional review board [study number: 19KHCC101], and exemption of consent was granted, compliant with the principles of the Declaration of Helsinki.

One-hundred sixty-six patients were included in the study; 143 patients were included in the analysis and 23 patients (14%) were excluded due to unknown CNS status (n-3) and CNS involvement at diagnosis (n-20). Patients were divided into two cohorts based on prior history of CRB. Cohort-1 had no CNS disease and received CRB (CNS−/CB+; n-110/143, 77%); and cohort-2 had no CNS disease, but did not receive CRB (CNS-/CB-; n-33/143; 23%). Patients’ characteristics were balanced in both cohorts with a few differences, such as younger patients in cohort 2 with more B-cell phenotype (table²). Here, we compare the incidence of post-alloHCT first CNS relapse between the two cohorts.

### Transplant characteristics

A total of 143 patients received TBI-based conditioning with either cyclophosphamide or fludarabine +/- thiotepa, with or without CRB. Patients were treated with either cyclophosphamide 60 mg/kg/day for two days (120mg/kg total), or fludarabine 30 mg/m2/day for three days (90mg/m^2^ total) (n=14) and/ or 10mg/kg thiotepa. TBI was delivered with parallel opposed fields (anterior and posterior) and extended source-surface distance using megavoltage therapy unit (Elekta Synergy) with 15 MV photons. The dose was prescribed to midplane and consisted of twice-daily fractions (200 cGy) to a total dose of 1200 cGy, with a 6-hour gap between fractions regardless of the age. Lung blocks were used during treatment to keep the median lung doses to 800-850 cGy.

**Figure 1.**
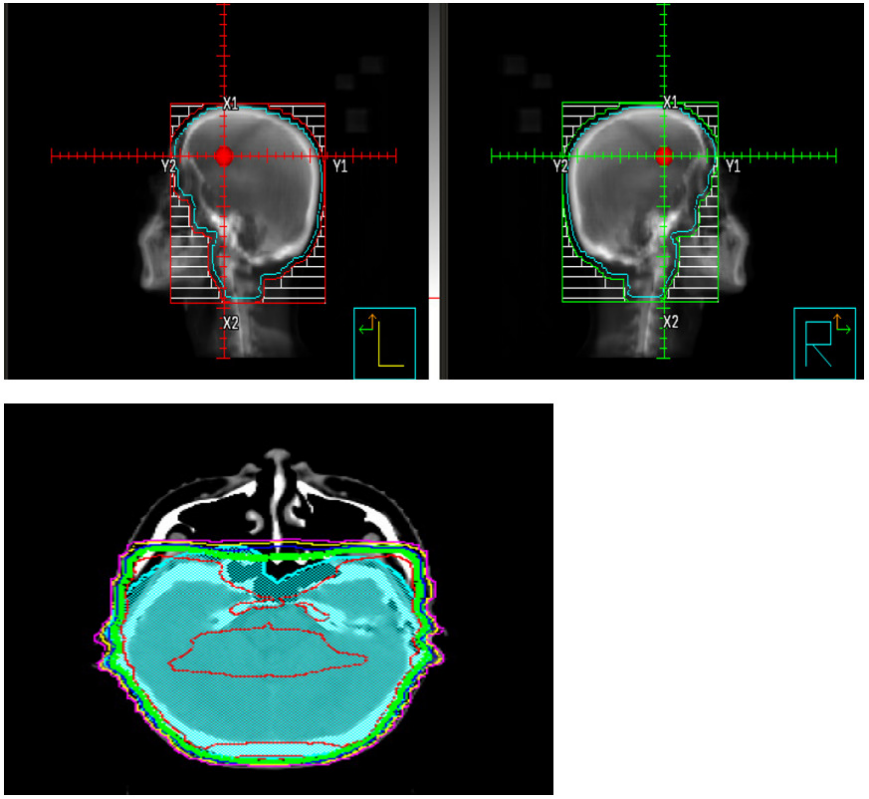
CRB and isodose line distribution (CRB: Cranial radiation boost) (CRB: cranial radiation boost; PBSC: peripheral blood stem cells, BM: bone marrow, CB: cord blood).

For CRB, CT simulation was performed using head mask; radiation fields consisted of two lateral opposing 6 MeV beams, including the whole skull content of brain and meninges. The anterior part was extended to include the meningeal cover of optic nerves up to the optic discs and down to the C2-C3 vertebral level. Shield was added to protect lens and oral cavity. This was a 95% coverage of the planning target volume (PTV) with homogenous isodose lines (figure-1). CRB was completed within two weeks prior to initiation of TBI. The CRB dose consisted of 12.6 cGy over 7 fractions by 3D conformal radiotherapy regardless of age, not to exceed 30cGy with prior cranial radiation. Three patients received a reduced dose of CRB of 8cGy. Graft-*versus*-host disease (GvHD) prophylaxis included either cyclosporine or tacrolimus and mycophenolate mofetil or methotrexate. ATG 2.5mg/kg was given in mismatched related donor transplants (MMRD) and PTCy 50mg/kg on day+3 and day+4 in haploidentical alloHCT.

### Statistical Analysis

Patients-disease and transplant-related characteristics were summarized using descriptive statistical methods. Median follow-up time was estimated using the reverse Kaplan-Meier (KM) method. Relapse-free survival (RFS) was defined from the date of alloHCT to the date of leukemia relapse, and overall survival (OS) was defined from the date of alloHCT to the date of death from any cause. The probabilities of RFS and OS were estimated using the KM method through 3 years after allotransplant.^16^ A Cox proportional hazard regression model was used to estimate the adjusted survival curves. CNS relapse was defined as occurrence of leukemic blasts in cerebrospinal fluid, contrast-enhancing brain or spinal and orbital lesion(s) on imaging attributed to leukemic involvement, with clinical symptoms consistent with CNS relapse. For the purposes of this study, CNS relapse was restricted to patients in whom the CNS was the first site of post-transplant relapse. The rationale for this definition was to exclude cases in which the CNS was a secondary relapse site following bone marrow relapse, a scenario that pre-transplant cranial irradiation could not have prevented.

Prognostic variables associated with CNS relapse, RFS and OS, were calculated using proportional hazards in univariate and multivariate analysis, including, patient’s cohorts (cohort-1 versus cohorts-2, CRB (yes versus no), remission status at alloHCT (CR1 versus ≥ CR2), thiotepa containing conditioning (Yes versus no), age at alloHCT (<18 year versus ≥ 18 years), ALL phenotype (T-cell versus B-cell), and disease risk (high-risk verdus standard risk).^15^ Two-sided P values of <0.05 was considered significant. All statistical analyses were implemented using Statistical Analysis System software version 9.4 (SAS Institute Inc., Cary, NC).

## Results

Patient-disease and transplant-related characteristics of both cohorts were detailed in tables 1 and 2. Sixty-seven (47%) patients were adults and 105 (73%) were males. Median age at transplantation was 18 (range: 3 –51 years). Ninety-five (66.4%) patients had B-cell phenotype and 45 (31.4%) had T-cell ALL. The Philadelphia (Ph) chromosome was identified in 34 patients (24%). Sixty-three (44%) patients received alloHCT while in CR1, and 80 (56%) in second remission or beyond. One hundred and thirty-one (92%) patients received myeloablative conditioning (MAC). The donor was matched-sibling related (MRD) in 125 patients (87 %), and 123 patients (86%) received blood graft. One hundred and ten patients received CRB (77%), while 33 (23%) did not (15 pediatric and 2 adults) based on physician’s preference. No patients received post-transplant prophylactic intrathecal chemotherapy.

**Table 1.** Patient-disease and transplant-related characteristics (N-143)

Variable	Value	Total N (%)
Age at alloHCT	Median and range	18y (3-51y).
White blood cells at diagnosis	Median and range x10E9/L	35 (2-7)
Gender	Female Male	105(73%) 38(26.5%)
Adults/Pediatrics	Adult pediatric	67(47%) 76(33%)
ALL subtype (T-cell/B-cell)	T-ALL B-ALL	46(33.5%) 95(66.5%)
Philadelphia by RT-PCR	Positive	34(24%)
Disease risk	High standard	48(34%) 95(66%)
Testicular boost	Yes	67(47%)
Disease status at alloHCT	CR1 ≥ CR2	63 (44%) 80 (56%)
Post all-HCT molecular only relapse by RT-PCR	Yes	2(1.3%)
Post alloHCT isolated CNS relapse	Yes	1(6.6%)
Conditioning regimen	Myeloablative Reduced Intensity	131(91.6%) 12(8.4%)
Thiotepa containing conditioning	Yes	6(4%)
Stem Cell Source	Blood graft	123(86%)
aGVHD	Yes	74(45%)
cGVHD	Yes	69(41%)
Disease status at last follow up	Remission	128(90%)
Patient status at last follow up	Alive	109(76%)

**Table 2.** Univariate and Multivariate analysis for first CNS post alloHCT relapse

Variable		Total	Cohort 1 (n-110) (CNS Neg/CB Pos)	Cohort 2 (n-33) (CNS Neg/CB Neg)	P-value	95% confidence interval	P-value
Gender	Female	39	28(25%)	27(25%)	0.330		
	Male	104	82(75%)	82(75%)			
Age at transplantation	Age at trans < 18	71	43(38.5%)	29(89%)	0.0001		
	Age at trans ≥ 18	71	67(61.5%)	4(11%)		3.59-45.84	<0.0001
ALL cell of origin (T-cell/B-cell)	B-ALL T-ALL	100 36	70(63%) 30(37%)	31(94%) 2(6%)	0.001	1.64-104.36	0.0151
Philadelphia chromosome	Positive	35	33(28%)	32(28%)	0.242		
	Negative	101	77(72%)	77(72%)			
Disease status prior to allo-HCT	CR1 CR≥2	69 73	59(52%) 52(48%)	58(52%) 52(48%)	0.358		
Stem Cell Source	Bone marrow	9	8(7%)	8(7%)	0.613		
	Cord blood	6	4(4%)	4(4%)			
	Peripheral blood	127	98(89%)	97(89%)			
BMT year	2007-2016	68	54(49%)	53(49%)	0.749		
	2017-2020	74	56(51%)	56(51%)			
Thiotepa-containing Conditioning	No Yes	140 2	107(98%) 3(2%)	107(98%) 2(2%)	1.000		
Risk stratification	Unknown risk	46	38(35%)	38(35%)	0.084		
	High risk	41	35(31%)	34(31%)			
	Standard risk	55	37(34%)	37(34%)			

### Relapse

Eleven percent of patients (n=15) experienced relapse after alloHCT. Extramedullary relapse with or without marrow involvement was the most common site (93%, n=14), with 4 relapses combined with testicular involvement and two with CNS involvement. There were 11 relapses (10%) in cohort-1 and 4 (12%) in cohort-2. Four patients had combined medullary and CNS involvement (26.6%), while three (20%) had combined marrow, CNS, and visceral involvement. Only one patient experienced isolated first post-alloHCT CNS relapse (6.6%). Age at transplant and phenotypic subtype were predictive for the first CNS relapse after alloHCT (see Table 2).

### Survival

**Figure 2.**
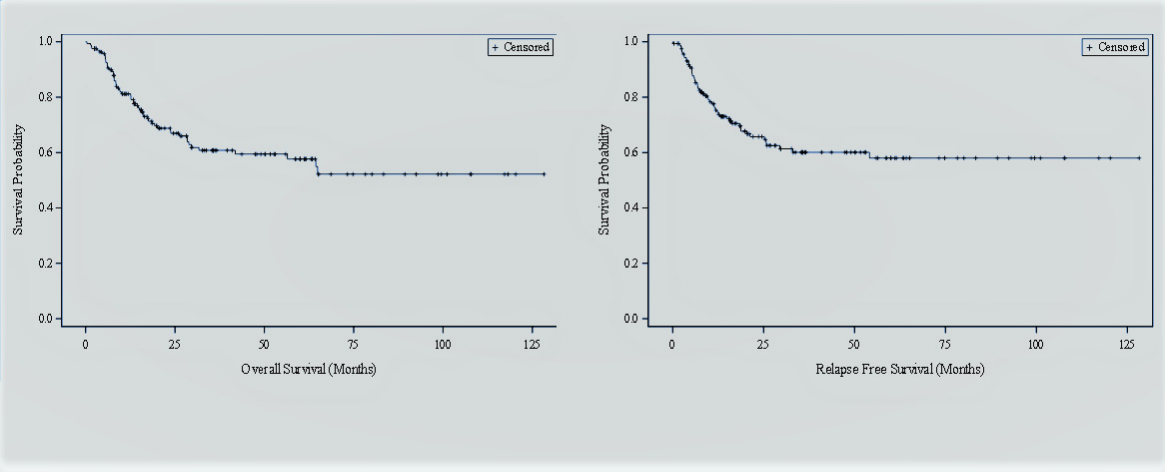
Overall (OS) and Relapse Free survival (RFS) following alloHCT for the whole group (n-143)

The median follow-up of survivors was 30 months for the entire group (range: 3-128); 30 months (3-60 months) for cohort-1 and 34 months (range: 3-128 months) for cohort-2. Seventy-six percent of patients (n=109) were alive at the last follow-up. The estimated 3-year OS was 61% (95% CI: 53-69), and RFS was 60% (95% CI: 52-69) for the entire cohort (figure 2). No significant difference was found in OS (P=0.73) or RFS (P=0.69). The estimated 3-year OS for cohort-1 (CNS-/CB+) was 66% (95% CI: 56, 75) compared with 54% (32, 75) for cohort- 2 (CNS-/CB-); P=0.73), Figure 3A. The Estimated 3-year RFS in both cohorts were 65% (95% CI: 54, 74) and 58% (95% CI: 38-77), respectively (P=0.69), Figure 3B. The estimated 3-year CNS-RFS was 99% and 100% in cohort 1 and cohorts with non-significant P value (P=0.595).

**Figure 3A.**
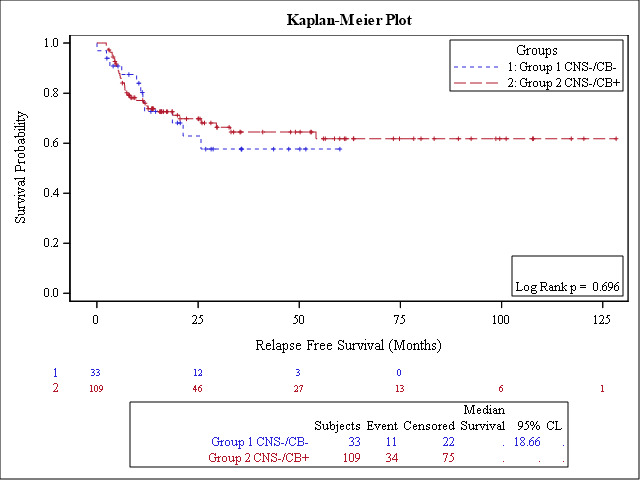
The estimated 3-year OS for cohort-1 (CNS-/CB+) was 66% (95% CI: 56, 75) compared with 54% for cohort- 2 (CNS-/CB-); P=0.73).

**Figure 3B.**
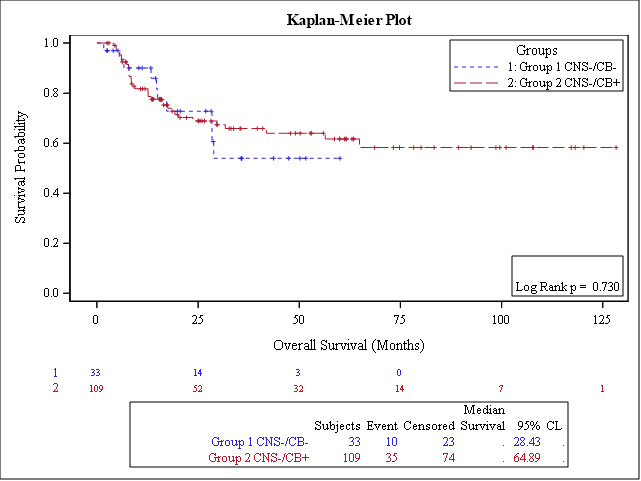
Estimated 3-year CNS-relapse free survival is 99% and 100% in cohort 1 and cohorts with non-significant P value (P-0.595).

## Discussion

Our study was to determine the effect of pre-transplant CRB on first CNS relapse post-alloHCT and survival outcomes in patients with ALL. Our results suggest there is no OS, RFS or CNS-RFS difference between patients with ALL with no CNS involvement at diagnosis, who received or not alloHCT prior CRB. The cumulative incidence of relapse was 10% in cohort-1 and 12% in cohort-2. One isolated first CNS relapse after alloHCT in cohort 1 compared to zero in cohort 2 (P-0.59).

Published data have been conflicting concerning the impact of CRB on posttransplant outcomes (see Table 3). Our results concur with those published by Gao et al, which showed no significant difference after the addition of CRB in patients with no CNS disease at diagnosis, but rather a benefit in patients with history of CNS involvement.^17^ On the other hand, our results contradict the conclusions of the report published by Justin et al, which showed an improved actuarial 7-year RFS (100% versus 76.4%; P=0.043) in favor of patients receiving CRB. Notably, this can be explained by the inclusion of high-risk patients in their cohort.^18^ Therefore, the current body of evidence in the literature is conflicting, because of several reasons, such as small sample size in different studies, heterogeneous group of patients (children and adults), lack of reporting on acute and long-term adverse effects and the presence of multiple confounding factors.^18^ Other reports suggested that CRB does not affect RFS and OS, which is similar to large data previously published.^12,18^ In light of this literature divergence, our study supports the idea of sparing patients with no CNS involvement at diagnosis from CRB and its acute and long-term toxicities.^17–19^ In addition, to CRB should be considered in patients with history of CNS involvement at diagnosis (CNS+/CRB+RT; n=20) as no patients relapsed in the CNS (data not shown).^19^ Age at alloHCT and phenotypic subtype of ALL were predictive for first CNS relapse after alloHCT,with respective P-values (P=0.001) and P=0.015, which may be used in patient’s selection for future studies (Table 2)=

**Table 3.** Published literature of the use of cranial radiation boost in ALL prior to alloHCT

**Study reference**	**Transplant type**	**Treatment groups**	**All Relapse**	**Post-transplant CNS relapse**	**OS**	**DFS**	**Conclusion**
Gao RW **et al 2016(n-213)**	MRD/MUD/CBT CR1	Total relapse CNS−/CRB−(n-160) CNS+/CRB+(n-11/30) CNS+/CRB-(n-11) CNS + PriorRT (n-12)	N-63 43/160; 26.9% 2/30; 6.7% 5/11; 45.5% 3/12; 25.0%	7(1.7%) n-4(2.5% n-2(6.6% n- 1(9%) N-Zero	2-yr OS 60% (95%CI: 53%- 67%) P-0.81	2-yr 54% (95% CI, 47%-61%)	No benefit of CRB in patients with no CNS disease, Benefit in patients with history of CNS involvement
**Sue William et al 2016(n-55)**	Allo-HCT-CR1 High risk	CRB group (n-36) No CRB group (n-19)	n-13 10 n-3	n-3 n-1 n-2	3-yr CNS free OS 63% 3-yr CNS free OS 82%	3-yr CNS free DFS 95% 3-yr CNS free DFS 52% 7-yr CNS RFS (100% vs 76.4% P-0.043)	Benefit of CRB in CNS -/CRB+.
Famoso Justin M **et al 2019(n-58)**	Allo-HCT High risk	CRB group No CRB group			7-yr OS 49.4% vs 43.5%; P = 0.921	7-yr PFS 78.3% vs 62.5%; P = 0.076) 7-year CNS RFS (100% vs 76.4% P = 0.043)	Benefit of CRB in CNS -/CRB+. high risk patients

### Study limitations

We acknowledge the limitations of our study, being retrospective and based on a relatively small sample size and heterogeneous population including children and adults, rarity of CNS events (relapses), several other confounding factors and the decision to administer CRB being based on the discretion of the treating physician, without toxicity data reporting.

In conclusion, in patients with ALL with no prior CNS involvement receiving their first alloHCT, augmenting TBI with CRB did not translate into improved survival or risk reduction of CNS relapse. Our results need to be validated in a larger and multi-institutional studies.

### Study approval statement

The study approved and the consent granted exemption by the Institutional Review Board (IRB) of King Hussein Cancer Center

### Conflict of interest

The authors have no conflicts of interest to declare

### Competing Interests

The authors did not receive any financial or nonfinancial support from any organization for the submitted work

### Data sharing

All data generated or analyzed during this study are included in this article

### Data availability

The data that support the findings of this study are openly https://doi.org/10.1016/j.prro.2018.12.005 1879-8500; Omura GA, Moffitt S, Vogler WR, Salter MM. Combination chemotherapy of adult acute lymphoblastic leukemia with randomized central nervous system prophylaxis. Blood. 1980 Feb; 55(2):199-204. PMID: 6928104.

### Authors Contributions

Khalid Halahleh contributed to the conception, design of the study, and interpretation of results, wrote the first draft and final manuscript. Mohamad Makoseh, Fawzi Abuhijlih, Lubna Ghatasheh, Rozan Al Far and Isra Muradi contributed to the data acquisition. Abdelatif Almousa drafted the TBI paragraph, Ramiz Abu-Hijlih wrote the abstract, CRB draft and acquision of isodense lines distribution figure and Hasan Hashem edited the manuscript. All authors approved the final manuscript.
